# Effect of exercise on improving myocardial mitochondrial function in decreasing diabetic cardiomyopathy

**DOI:** 10.1113/EP091309

**Published:** 2023-10-16

**Authors:** Feng Zhang, Jian jian Lin, Hao nan Tian, Jun Wang

**Affiliations:** ^1^ Sports Physiology Department Beijing Sport University Beijing China; ^2^ PE Teaching and Research Office University of International Relationship Beijing China

**Keywords:** DCM, diabetic cardiomyopathies, exercise, myocardial mitochondria

## Abstract

Diabetic cardiomyopathy (DCM) is a significant cause of heart failure in patients with diabetes, and its pathogenesis is closely related to myocardial mitochondrial injury and functional disability. Studies have shown that the development of diabetic cardiomyopathy is related to disorders in mitochondrial metabolic substrates, changes in mitochondrial dynamics, an imbalance in mitochondrial Ca^2+^ regulation, defects in the regulation of microRNAs, and mitochondrial oxidative stress. Physical activity may play a role in resistance to the development of diabetic cardiomyopathy by improving myocardial mitochondrial biogenesis, the level of autophagy and dynamic changes in fusion and division; enhancing the ability to cope with oxidative stress; and optimising the metabolic substrates of the myocardium. This paper puts forward a new idea for further understanding the specific mitochondrial mechanism of the occurrence and development of diabetic cardiomyopathy and clarifying the role of exercise‐mediated myocardial mitochondrial changes in the prevention and treatment of diabetic cardiomyopathy. This is expected to provide a new theoretical basis for exercise to reduce diabetic cardiomyopathy symptoms.

## Abstract (highlights)

- **What is the topic of this review?**
  The mechanism of exercise decrease diabetic cardiomyopathy by improving the mitochondrial homeostasis of the myocardium, and new insights for the treatment of diabetic cardiomyopathy with exercise.
- **What advances does it highlight?**
  Mitochondrial dysfunction induced by a high‐glucose environment is one of the main causes of diabetic cardiomyopathy. The mitochondrial mechanism is mainly mitochondrial metabolic substrate disorder, mitochondrial dynamics change, mitochondrial Ca^2+^ regulation imbalance, and so on. Exercise can improve the function of myocardial mitochondria in multiple ways, so as to achieve the effect of decreasing diabetic cardiomyopathy.

## 1 INTRODUCTION

With changes in people's dietary habits and reductions in physical activity, the global growth rate of the number of patients with diabetes has been increasing year by year. Statistics show that the global number of people living with diabetes in 2019 was approximately 463 million, and this is expected to increase to 578 million in 2030 and 700 million in 2045 (Saeedi et al., 2019). Damage to the cardiovascular system is one of the main manifestations of organ damage and also the primary cause of mortality in diabetic patients. Studies have shown that over 50% of patients with diabetes die or are disabled as a result of cardiovascular complications (Teodoro et al., 2019). Among these, diabetic cardiomyopathy (DCM) refers to myocardial structural abnormalities and dysfunction that are different from hypertension, major valvular diseases and coronary artery diseases. The clinical course of DCM is unique and its pathogenesis is complex. Various mechanisms play a role in the occurrence and development of DCM, including hyperglycaemia, insulin resistance, impaired glucose and lipid metabolism, mitochondrial dysfunction, oxidative stress, deposition of extracellular matrix, and apoptosis (Gjia et al., 2018). Furthermore, these are associated with early diastolic and later systolic dysfunction, ultimately leading to cardiac failure (Ketenci et al., 2022). In addition, the course of DCM is slow, and it is often diagnosed when there is some degree of dysfunction in the heart, which is a major cause of death in patients with diabetes (Ma et al., 2021). Cardiomyocytes have high energy requirements and produce adenosine triphosphate (ATP) mainly through oxidative phosphorylation (OXPHOS) to maintain normal systolic function, so they are rich in mitochondria (Liao et al., 2020). The mitochondria in the myocardium make up 35–40% of the cytoplasmic volume, and provide 95% of the ATP for heart beating; consequently, mitochondria are essential organelles in cardiomyocytes (the ‘power plants’ of the cell). It is inevitable that damage to myocardial mitochondrial function will lead to impairment of cardiac function (Wang et al., 2023; Zhang et al., 2023). Previous clinical and animal experiments have confirmed that mitochondrial dysfunction plays a vital role in the pathological progression of DCM (An & Rodrigues, 2006; Boudina & Abel, 2006). Abnormalities and structural disorders of mitochondria are evident in several diabetic models and are closely related to reduced cardiac ATP production, impaired ejection fraction and the rate of short‐axis shortening (Wang et al., 2015). Currently, the main treatment for DCM is glycaemic control, so as to reduce the morbidity of cardiovascular disease in patients with diabetes, but the treatment is unsatisfactory (Kirwan et al., 2017). However, numerous clinical studies have shown that a variety of exercises, such as water sports, Nordic walking, self‐made aerobics, yoga, pilates, tai chi and dance, have a positive impact on the recovery from diabetes and DCM (Cugusi et al., 2019, 2017, 2019, 2017; Nuhu & Maharaj, 2018; Rees et al., 2017; Thind et al., 2017; Zhou et al., 2019). At the same time, exercise can effectively improve the structure and function of myocardial mitochondria, thereby enhancing cardiac function. Therefore, we speculate that exercise may prevent the onset of DCM by improving the functioning of myocardial mitochondria.

## 2 MITOCHONDRIAL PATHOGENESIS OF DIABETIC CARDIOMYOPATHY

**FIGURE 1:**
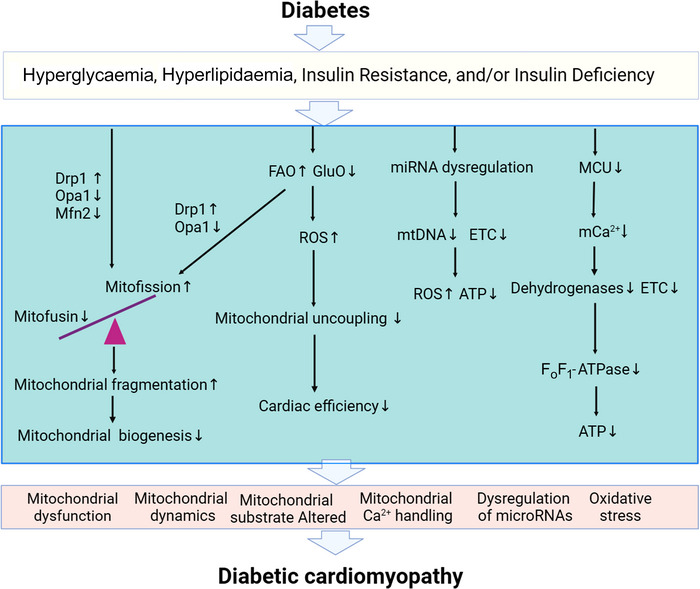
Mitochondrial pathogenesis mechanism of diabetic cardiomyopathy. ATP, adenosine triphosphate; Drp1, dynamic‐related protein1; ETC, electron transport chain; FAO, fatty acid oxidation; F_O_F_1_‐ATPase, ATP synthase holoenzyme; GluO, glucose oxidation; MCU, mitochondrial calcium uniporter; Mfn2, mitochondrial fusion protein 2; mtDNA, mitochondrial DNA; Opa1, optic atrophy protein; ROS, reactive oxygen species. Created using BioRender.com.

Glucose and lipid metabolic disorders can cause changes in mitochondrial structure and function. In the myocardial tissue of DCM mice, mitochondria are disordered and swollen, mitochondrial cristae are broken, and some mitochondria are vacuolated. Compared with healthy mice, the formation of autophagosomes in the myocardial tissue of DCM mice is decreased, reducing the level of mitophagy. The above changes in mitochondria are related to a decrease in mitophagy mediated by the PTEN‐induced kinase 1 (Pink1)/parkin pathway (Wang, 2015). In a study of diabetic animal models, myocardial mitochondrial content and oxidative phosphorylation‐related components were decreased, as well as mitochondrial oxidative phosphorylation capacity (Boudina et al., 2005; Lashin & Romani., 2004). These changes may lead to a decrease in the ability of myocardial mitochondria to synthesize ATP and thereby reduce the supply of energy available for myocardial cell contraction, and ultimately progress to myocardial dysfunction. In addition to the changes mentioned above in myocardial mitochondrial structure and function, other mitochondrial factors are associated with the pathogenesis of DCM as well (Figure 1).

### 2.1 Disorder of mitochondrial metabolic substrates

The cardiomyocyte must consume a large amount of energy to maintain the pumping of the heart, accounting for about 8% of the body's ATP consumption (Gollmer et al., 2020). In normal myocardium, 60–70% of ATP is derived from the oxidation of fatty acids, and only a small portion (about 20−30%) comes from the oxidation of glucose, lactic acid, ketone bodies and amino acids (Bertero & Maack., 2018; Gertz et al., 1988). Glucose transporters (GLUTs) play a key role in transmembrane transport and uptake of glucose, while pyruvate dehydrogenase (PDH) is a key enzyme in regulating glucose metabolism: in the diabetic state, the activation or expression of GLUTs and PDH is reduced due to insulin resistance and other factors (Tian et al., 2018). This leads to a decrease in myocardial glucose‐based energy supply as the proportion of energy supplied by fatty acids increases (Tian et al., 2018). Compared with glucose, fatty acids as a substrate for energy metabolism require about 12% more oxygen to produce the same amount of ATP; the ATP/oxygen efficiency ratio of fatty acids is lower than that of glucose with the same capacity (Tian et al., 2018). Alterations in mitochondrial metabolic substrates increase the amount of oxygen uptake and increase its capacity load. Long‐term changes will lead to a decline in mitochondrial function. For example, developing insulin resistance and type 2 diabetes may enhance the uptake of free fatty acids (FFA) by cardiomyocytes. Excessive intake of fatty acids, in turn, can aggravate damage to mitochondrial fatty acid β‐oxidation, leading to mitochondrial dysfunction and accumulation of lipotoxic metabolites in the patient's myocardium; this can lead to cardiac dysfunction (Chen et al., 2022).

In DCM, increased fatty acid oxidation ratios may be responsible for increased activity of peroxisome proliferator‐activated receptors (PPARs), which are key proteins that regulate glucose and lipid metabolism and play an important role in the pathogenesis of cardiac dysfunction and diabetes (Wu & Wan, 2017), and fatty acid‐induced PPAR activation leads to up‐regulated expression of genes encoding various proteins and enzymes involved in cellular fatty acid utilization (Glatz & Luiken, 2017). Fatty acids activate PPARα and peroxisome proliferater activated receptor γ coactivator 1‐α (PGC‐1α). This increases PPARα binding to PPAR response elements on promoters of genes which encode proteins related to the uptake and oxidation of fatty acids, including carnitine palmitoyltransferase1 (CPT1) and long‐chain acyl‐CoA dehydrogenase (LCAD), thereby enhancing mitochondrial uptake and the oxidation of fatty acids (Barger & Kelly, 2000). Indeed, cardiomyocyte‐specific overexpression of PPARα results in increased expression of fatty acid utilisation genes, leading to increased fatty acid oxidation. Reduced expression of genes involved in glucose oxidation leads to reduced glucose uptake and oxidation (Finck et al., 2002). The change in the above process reduces the proportion of mitochondrial glucose oxidation and increased the proportion of fatty acid oxidation.

### 2.2 Changes in mitochondrial dynamics

Mitochondria are highly dynamic organelles, and their number, morphology and function are maintained by continuous fusion and division. This process of dynamic balance is called mitochondrial dynamics (Willems et al., 2015). The dynamic stability of mitochondria is a vital prerequisite for ensuring the standard structure and amount of mitochondria and for the normal function of the mitochondria (Chen et al., 2021). Mitochondrial fusion protein 1/2 (MFN1/2) and optic atrophy protein 1 (OPA1) are two of the proteins shown to control primarily the fusion of mitochondria. MFN1/2 is localised to the outer membrane of the mitochondria and mediates the contact between adjacent mitochondria and the outer membrane during fusion (Chen et al., 2003). OPA1 is located on the mitochondrial inner membrane and mediates mitochondrial inner membrane fusion (Zorzano et al., 2010). Dynamic‐related protein 1 (Drp1) and mitochondrial fission 1 (Fis1) are involved in the completion of mitochondrial division. Fis1 is located on the outer membrane of the mitochondria and recruits Drp1 in the cytoplasm to the outer membrane of mitochondria, forming a ring that gradually tightens, completing the mitochondrial division process (Chan, 2006).

Mitochondrial fusion–fission is a critical process to maintain the quantity and quality of mitochondria in the myocardium (Zheng & Qu, 2022). In contrast, in patients with DCM, an increase in blood glucose levels leads to an imbalance in mitochondrial dynamics. The activity of mitochondrial fission protein is higher than that of fusion protein, which promotes mitochondrial fragmentation, thereby weakening the OXPHOS of the mitochondrial respiratory chain and increasing the concentration of reactive oxygen species (ROS), mitochondrial membrane potential (MMP) depolarization, and mitochondrial DNA (mtDNA) abnormalities, and eventually leading to DCM (Weng et al., 2017; Wu et al., 2019). Up‐regulation of the expression of the mitochondrial fission protein Drp1 leads to mitochondrial dysfunction and insulin resistance. In contrast, down‐regulation of Drp1 expression can prevent hydrogen peroxide‐induced myocardial mitochondrial dysfunction. These mechanisms may be related to Drp1 regulation of mitochondrial ROS generation, amelioration of changes in membrane potential and ATP generation (Watanabe et al., 2014). Studies have shown that in myocardial tissue of DCM rats, the expression of mitochondrial fission protein Drp1 is significantly higher than in the non‐diabetic group, while the fusion proteins MFN1 and MFN2 are lower. As a result, mitochondrial fission in cardiomyocytes progressively increased, and fusion and biogenesis decreased (Yu et al., 2021). This suggests that DCM displays disordered mitochondrial dynamics, as well as increased fission and decreased fusion. Mitochondrial fusion and division are always in a dynamic equilibrium. Balancing the two against DCM remains essential in modern medical research.

### 2.3 Imbalance in mitochondrial Ca^2+^ regulation

The main feature of DCM is diastolic and systolic dysfunction, and Ca^2+^ circulation plays a crucial role in the contraction and relaxation of cardiomyocytes (Lin et al., 2023). Ca^2+^ is an intermediate messenger in the excitation–contraction‐coupling mechanism. Normal Ca^2+^ concentration in cardiomyocytes is an important guarantor of the maintenance of heart function, but when Ca^2+^ uptake ability is disordered, this will lead to mitochondrial dysfunction. Studies in animal models of DCM have demonstrated that a disorder of Ca^2+^ regulation of the mitochondria of the myocardium is a specific disease (Belke et al., 2004). In the diabetic mouse model, there was an increase in resting Ca^2+^ concentration in the cardiomyocytes, an extension of the Ca^2+^ influx time, a decrease in the difference between the intracellular and extracellular Ca^2+^ concentration, reduced responsiveness to extracellular Ca^2+^ changes, and a decrease in the activity of the calcium pump in the sarcoplasmic reticulum leading to an impairment of Ca^2+^ reuptake from the stromal network (Li et al., 2006). Mitochondria produce ATP while storing Ca^2+^, a crucial intracellular Ca^2+^ buffer. Intracellular Ca^2+^ primarily enters the mitochondrial matrix through the mitochondrial calcium uniporter (MCU) located on the inner membrane of the mitochondrion. Under normal physiological conditions, the mitochondrion requires only a tiny amount of change in Ca^2+^ concentration to activate some important dehydrogenases such as F_o_F_1_‐ATP and to promote ATP production.

Indeed, it has been suggested that abnormal expression of ryanodine receptor 2 (RyR2), sarcoplasmic reticulum Ca^2+^‐ATPase and Na^+^/Ca^2+^ exchange protein in myocardial cells of diabetic rats may partly lead to cardiac dysfunction (Zhao et al., 2014). Mitochondria are very sensitive to the Ca^2+^ concentration, and if it is either too high or too low this will affect the mitochondrial OXPHOS process. Diaz‐Juarez et al. (2016) found that high‐glucose stimulation of cardiomyocytes led to a decrease in MCU expression, a decrease in mitochondrial Ca^2+^ concentration, and perturbed profiles of glucose and lipid metabolism. Mitochondrial Ca^2+^ overload leads to intracellular oxidative damage, and the two have reciprocal causation, forming a vicious cycle, ultimately leading to apoptosis or necrosis, thus affecting the systolic and diastolic function of the heart (O‐Uch et al., 2014). MCU and mitochondrial calcium uptake protein 1 (MICU1) have been shown to play a significant role in the transport and uptake of Ca^2+^. Mice with streptozotocin (STZ) diabetes showed increased expression of MICU1 in the heart but decreased expression of MCU and MCU regulatory factors (such as EMRE, an MCU subunit), leading to mitochondrial Ca^2+^ uptake, reduced mitochondrial function and decreased heart function. Restoring MCU can rescue both cardiac and mitochondrial respiratory dysfunction, which emphasizes the important role of mitochondrial Ca^2+^ restoration in the treatment of cardiac dysfunction (Suarez et al., 2018; Dillmann, 2019). However, the mitochondrial permeability transition triggered by Ca^2+^ overload likely complicates this therapeutic strategy (Crompton et al., 1988; Griffiths & Halestrap, 1991). Meanwhile, overexpression of MICU1 in the myocardium of diabetic patients can activate the antioxidant system, reduce myocardial fibrosis and cardiac hypertrophy, and partially prevent the development of DCM. However, it should be noted that increased Ca^2+^ uptake via MICU1 overexpression mitigates heart failure in DCM models but leads to increased mortality in non‐diabetic mice (Ji et al., 2017). Based on the studies cited above, it has been shown that Ca^2+^ homeostasis plays a vital role in resistance to DCM. Meanwhile, the role of the same molecule or pathway in different experimental models should be considered.

### 2.4 Alteration of mitochondrial oxidative stress

Oxidative stress is a state of relative non‐equilibrium caused by the excessive generation of ROS (superoxide radicals, hydrogen peroxide, hydroxyl radicals and other substances; Wilson et al., 2018) or inadequate antioxidant capacity within the body. Under physiological conditions, mitochondria generate ATP through OXPHOS to provide energy for cells. The process of electron reduction in the respiratory chain results in the reduction of oxygen to ROS (Scialò et al., 2020). In contrast, excessive blood glucose leads to increased production of ROS and insufficient antioxidant capacity, which will promote the accumulation of oxygen free radicals in the heart, thereby promoting oxidative stress of cardiomyocytes and leading to excessive apoptosis (Frustaci et al., 2000; Kaludercic & Di Lisa, 2020). ROS derived from mitochondria play an important role in regulating cellular physiological functions. However, in the diabetic state, metabolic changes such as hyperglycaemia, insulin resistance and high levels of FFA lead to the accumulation of mitochondrial ROS in the cells, mitochondrial dysfunction, an enhanced inflammatory response, the formation of advanced glycation end products, and fibrosis of the fibroblasts, or pyroptosis of myocardial cells, ferroptosis and so on, ultimately leading to cardiac dysfunction and participating in the pathogenesis of DCM (Evangelista et al., 2019; Chen et al., 2020; Kaludercic & Di Lisa, 2020). Indeed, mitochondria, NO synthase, xanthine oxidase and NADPH oxidase are the significant sources of cardiac reactive oxygen molecules that have been shown to play a key role in the progression of DCM (Wilson et al., 2018). NADPH oxidase 4 (NOX4) is an enzyme that is the primary source of oxidative stress in heart failure (Kuroda et al., 2010). The excess superoxide generated by the metabolic abnormalities described above react rapidly and non‐specifically to DNA, protein, lipids and carbohydrate, promoting mitochondrial injury and leading to irreversible cytotoxicity (Forbes & Cooper , 2013).

In addition, long‐term disorder of glycolipid metabolism may lead to overgeneration and insufficient clearance of ROS. Mitochondria are the primary source of ROS production in diabetic patients. The generation of ROS by mitochondria can lead to mitochondrial dysfunction, which in turn aggravates ROS generation and forms a vicious cycle, leading to detrimental effects on cell function and survival status. Several studies have demonstrated that oxidative stress can promote apoptosis through mitochondria‐ and non‐mitochondria‐dependent pathways (Tian, 2022). One‐way mitochondria can cope with ROS damage is to induce apoptosis by increasing the permeability of the outer membrane of the mitochondrion, leading to a rapid influx of solutes and water into the mitochondrial matrix; collapse of the proton gradient, halting ATP synthesis; excessive uptake of Ca^2+^ by mitochondria; and ultimately complete impairment of mitochondrial function (Dia et al., 2020). The view expressed by Wang et al. (2021) was that mitochondrial oxidative stress plays an essential role in DCM pathogenesis, by activation of cellular fibrosis by mitochondrial ROS and apoptosis‐related pathways; promotion of mitochondrial energy uncoupling with reduced ATP production; promotion of apoptosis of cardiomyocytes; damage to mtDNA and its encoded proteins in such a way that myocardial cell function is impaired; increased mitochondrial inner membrane permeability, promoting calcium overload; and reduced level of mitophagy. The combination of various mechanisms leads to damage to myocardial cell structure and function, an increase of fibrosis, and the occurrence of DCM. At the same time, DCM will further aggravate mitochondrial oxidative stress. Therefore, we believe that, to a certain extent, the control of mitochondrial oxidative stress can effectively delay the development of DCM.

### 2.5 miRNA dysregulations

miRNAs are single‐stranded non‐coding RNA molecules of about 23 nucleotides in length. They can regulate their transcription or translation by inhibiting mRNA pairing, thereby playing a significant role in gene regulation in animals and plants (Hathaway et al., 2018). Indeed, it has been confirmed that dysregulation of many different miRNAs can lead to an increased likelihood of cardiovascular disease, including heart failure and DCM (Hathaway et al., 2018; Guo & Nair, 2017). For example, miRNA‐103, miRNA‐107, miRNA‐143, miRNA‐802 and miRNA‐181 have all been shown to have essential roles in the regulation of systemic glucose metabolism as well as insulin sensitivity, indicating that some miRNAs have a role in the pathogenesis of insulin resistance and type 2 diabetes (Zhou et al., 2014).

Mitochondrial dysfunction is partly due to changes in miRNA function and expression. For example, an increase in miRNA‐378 in myocardial mitochondria of STZ diabetic mice weakens the translation of the ATP6 subunit of F_o_F_1_‐ATPase (ATP synthase holoenzyme). It has a detrimental effect on the oxidative phosphorylation function of mitochondria in the myocardium (Chen et al., 2017). miRNA‐195 overexpression observed in type 1 (T1DM) and type 2 (T2DM) diabetes mellitus hearts may lead to down‐regulation of sirtuin 1 (SIRT1), and impaired SIRT1 activity may affect oxidative metabolism, mitochondrial function and increase ROS production (Zheng et al., 2015). The direct target of PGC‐1α is the miRNA‐29a gene. In STZ‐induced diabetic animals, the level of miRNA‐29a in the myocardium is reduced, which can contribute to the induction of PPARα and fatty acid oxidation (FAO) expression as well as the biogenesis of mitochondria (Diao et al., 2011). Baseler et al. (2012) found elevated levels of miRNA‐141 in T1DM mice, which could be detrimental to solute carrier family member 25 (Slc25a3) activity, thereby affecting inorganic phosphate supply and subsequent ATP regeneration. These studies have shown that miRNAs can interfere with the different pathways necessary for maintaining mitochondrial oxidative function and the roles of related proteins and enzymes. Therefore, abnormal regulation of miRNA may also be the main cause of mitochondrial dysfunction in DCM, but more studies are needed to further verify the exact mechanism, the process of interaction, and other possible miRNA features regulating mitochondrial function.

## 3 EXERCISE‐MEDIATED IMPROVEMENT IN MYOCARDIAL MITOCHONDRIAL FUNCTION DECREASE THE ONSET OF DIABETIC CARDIOMYOPATHY

**FIGURE 2:**
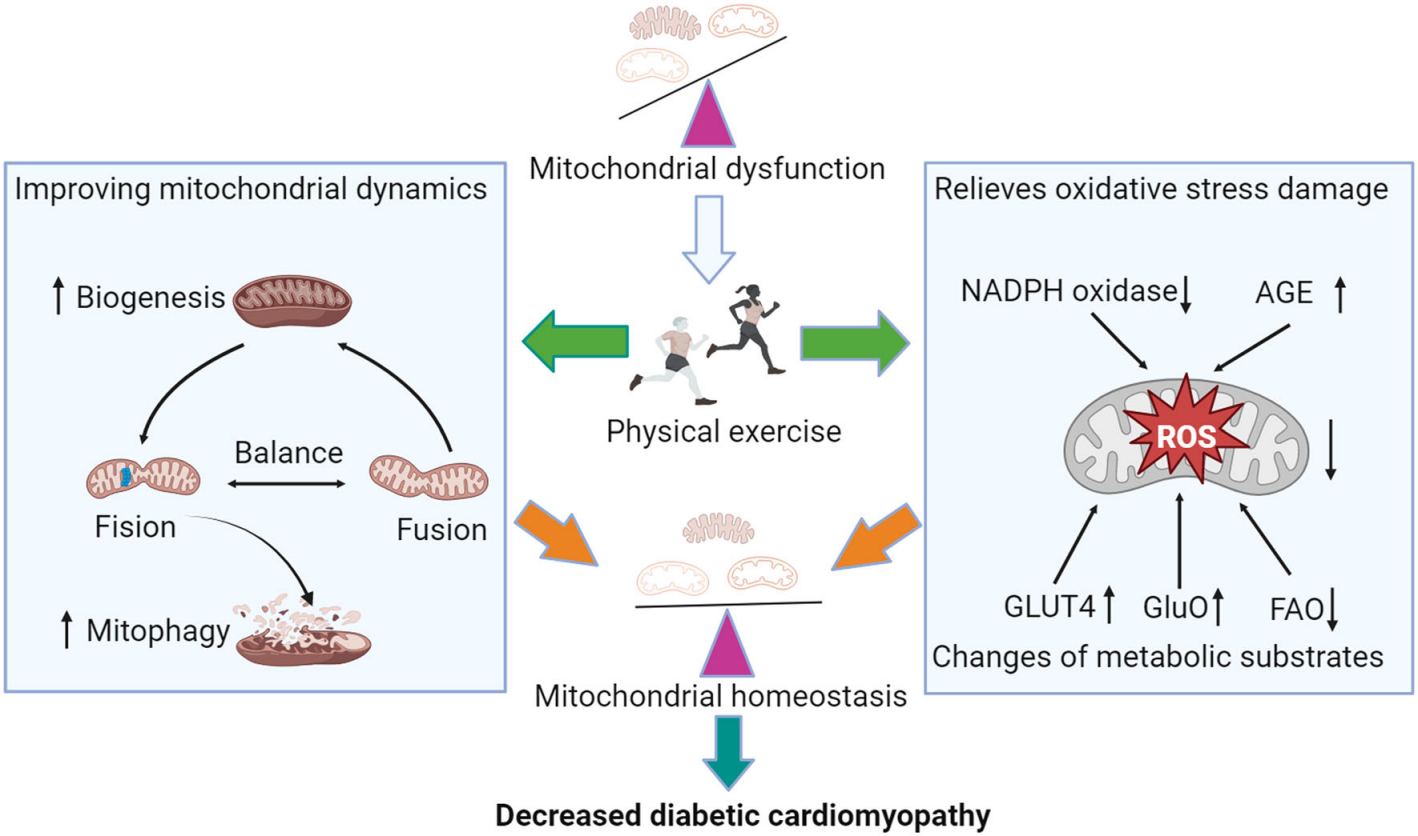
Mechanism of exercise improving myocardial mitochondria and decreasing diabetic cardiomyopathy. Exercise can promote biogenesis by balancing the process of myocardial mitochondrial fusion and fission, improving autophagy and oxidative stress levels, thereby optimizing myocardial mitochondrial homeostasis and playing a role in decreasing diabetic cardiomyopathy. AGE, advanced glycation end products; FAO, fatty acid oxidation; GluO, glucose oxidation; NADPH, nicotinamide adenine dinucleotide phosphate; ROS, reactive oxygen species. Created using BioRender.com.

Studies have confirmed that exercise training does not alter significant cardiac hypertrophy (including increased overall cardiac cross‐sectional area) in diabetic patients (Mali et al., 2014; Nath et al., 2017). However, it can reverse the disorder of the myocardial sarcomere and the arrangement of mitochondria in the diabetic mouse. For example, studies have shown that 6 weeks of exercise significantly improved the average cross‐sectional area, mitochondrial circumference, mitochondrial Feret's diameter and the inner mitochondrial membrane in mice with diabetes. At the same time, it also promotes the changes in mitochondrial morphology in diabetic mice, including mitochondrial membrane wrinkles, irregularities and decreased intimal ridges, thereby effectively alleviating the symptoms of DCM (Jin et al., 2022). These results prove that exercise can delay the onset of DCM by improving mitochondrial morphology and structural changes. In addition, exercise can also treat or improve DCM symptoms by improving myocardial mitochondrial function (Figure 2).

### 3.1 Exercise optimises mitochondrial quality control

Mitochondrial quality control plays an essential role in mitochondrial network homeostasis and mitochondrial function, mainly consisting of three processes: mitochondrial biogenesis, mitochondrial dynamics and autophagy. The myocardium can repair and remove damaged mitochondria, reduce the production of ROS and increase the occurrence of new mitochondria through the mitochondrial quality control process (Ma et al., 2020).

PGC‐1α is a multifunctional transcription coactivator and a marker of mitochondrial biogenesis (Fujimoto et al., 2011). Studies have shown that the mRNA expression of PGC‐1α in diabetic myocardium is decreased, and the expression levels of its downstream transcription factors such as nuclear respiratory factor 1 (NRF‐1), mitochondrial transcription factor A (Tfam) and mitochondrial transcription factor B2 (Tfb2m) are down‐regulated. Tfam is essential in mitochondrial DNA replication, transcription and nucleoid formation. Wang et al. (2015) have shown that mitochondrial DNA damage and mitochondrial biosynthesis in diabetic rats can be restored to a certain extent through exercise training; the mechanism is related to regulating mitochondrial biosynthesis and function by PGC‐1α by regulating the expression of its downstream transcription factors. In patients with DCM, long‐term moderate‐intensity exercise training reduces myocardial fibrosis and apoptosis through activation of the PGC1‐α and Akt signalling pathways; increases mitochondrial biosynthesis; and maintains normal myocardial function (Wang et al., 2015). In addition, Jin et al.’s (2022) latest study identified fibroblast growth factor 21 (FGF21)–sirtuin 3 (SIRT3) as a novel molecular sensor of exercise, which can promote mitochondrial integrity and stability of cardiac function in the diabetic myocardium， The expression of mitochondrial SIRT3 induced by FGF21 is a key intermediary to prevent DCM through exercise. This view supports the role of FGF21 as a stress response factor in cellular stress defence. Given the therapeutic potential of FGF21 analogues in metabolic disease, the selective activation of the FGF21 signalling axis in cardiomyocytes by exercise to induce SIRT3 may be an effective strategy for treating various heart diseases.

Vettor et al. (2014) found that exercise can both activate and promote the biosynthesis of mitochondria in the myocardium. Following 6 weeks of incremental load swim training (5 days a week, 90 min each time), the expression of endothelial nitric oxide synthase (eNOS) in the endothelium of rat myocardium can be increased, and the expression of PGC‐1α, NRF‐1 and Tfam can also be improved. At the same time, the number, volume and rate of glucose uptake of the mitochondria also increased significantly as a result of exercise. In another study, after a 5‐week moderate‐intensity treadmill exercise intervention in rat models of DCM, we found that the expression levels of markers of myocardial mitochondrial biogenesis, mitochondrial fusion protein 2 (Mfn2) and Drp1 were increased, which increased the number of mitochondria and improved their function (Veeranki et al., 2016). In addition, muscle training may also improve myocardial function and enhance the efficiency of myocardial mitochondrial work in mice with diabetes, which is related to the high expression of the mitochondrial protein PGC‐1α and Tfam (Ko et al., 2018). Veeranki et al. (2016) studied cardiac function in obese diabetic mice and found that endurance exercise training for 5 weeks on a treadmill improved the function of intercellular communication by increasing the protein expression of connexin43 (Cx43), thus improving cardiac ejection capacity. Conversely, it was also found that after an exercise intervention, there was no change in the level of the mitochondrial fusion protein‐related factor Mfn2 in the myocardium of diabetic mice. Still, exercise decreased the mitochondrial fission‐related protein Drp1 to normal levels, increasing the Mfn2/Drp‐1 ratio. The results of this study show that in the obese diabetic state, the process of mitochondrial division rises, reducing the regenerative capacity of the mitochondria and ultimately leading to a decrease in systolic cardiomyocyte function. However, exercise training can improve myocardial function by reversing this change.

As one of the important mechanisms of mitochondrial quality control, mitophagy can degrade and remove damaged/excess mitochondria in cells to maintain the normal life activities of the cells (Yao et al., 2021; Zhang et al., 2023). This autophagy process in which cells highly selectively degrade mitochondria can prevent the accumulation of abnormal mitochondria (Morales et al., 2020). In addition, appropriate mitophagy scavenges metabolic substrates, which are essential for cardiomyocyte metabolism under stress conditions (Pietzsch et al., 2020). Overexpression of mitophagy can significantly degrade mitochondria in cells, leading to mitochondrial damage, disordered cellular energy metabolism and cell death (Yi et al., 2022). Autophagy can selectively degrade damaged or senescent mitochondria and allow them to be repurposed (Bharath et al., 2021). Previous studies have shown that in the metabolic syndrome heart, mitophagy is either down‐regulated (Bharath et al., 2021) or up‐regulated (Russo et al., 2012). Tong et al. (2019) found that mitophagy was up‐regulated early in the course of DCM but did not increase the level of cardiac protection. Mitochondrial dysfunction can be alleviated by improving the function of mitochondrial autophagy and reducing lipid accumulation, thereby preventing diastolic heart dysfunction. Xie et al. (2011) activated the autophagy level using drugs (such as fenofibrate, metformin and resveratrol) to slow down diabetic‐induced cardiac dysfunction, while inhibiting autophagy can worsen the manifestation of diabetic cardiomyopathies. The above studies demonstrate that appropriately increasing the level of mitophagy can effectively reduce the impairment of cardiac function caused by diabetic cardiomyopathies. Proper exercise may have a similar effect. After 8 weeks of aerobic interval treadmill training (80–90% ${\dot{V}}_{O_{2}\max}$, 65–75% ${\dot{V}}_{O_{2}\max}$), there was an improvement in the function of myocardial mitochondria, which manifested mainly as an increase in the ADP/O ratio, increase in the activities of complex I, III and IV, increased mitochondrial fission, decreased fusion, increased expression of the nuclear protein PGC‐1α, decreased expression of the extracellular signal‐related kinase 1/2 (ERK1/2)–c‐Jun N‐terminal kinase (JNK)–P53 signalling pathway, and increased level of mitophagy (Jiang et al., 2014). After the same 8 weeks of aerobic exercise, the myocardial autophagy‐related AMP‐activated protein kinase (AMPK)–Unc‐51 like autophagy activating kinase 1 (ULK1) pathway was improved in spontaneously type 2 diabetic rats, leading to an increase in the level of mitophagy in the myocardium as well as effectively attenuating the pathogenesis of type 2 DCM (Hou et al., 2022). Too little or too much mitophagy is not conducive to its correct function. Exercise training regulates the level of mitochondrial autophagy, and to achieve the purpose of enhancing myocardial function, attention should be paid to the regulation of training variables, according to different conditions to develop reasonable exercise programs, in order to achieve the optimal level of mitochondrial autophagy.

### 3.2 Exercise improves myocardial mitochondrial metabolic substrates and oxidative stress levels

One of the most common symptoms of DCM is a change in the substrate of myocardial mitochondrial oxidation. It is also one of the drivers of generating ROS and oxidative stress. Hyperglycaemia is one of the leading causes of these detrimental adaptations, including impaired insulin signal transduction leading to the consumption of the glucose transporter (GLUT4) in the blood, which results in a change in FFA oxidation, activation of protein kinase C (PKC), up‐regulation of the polyol and hexosamine pathway, and the generation of advanced glycation end products as well as ROS (Doliba et al., 2018; Jia et al., 2018; Singhet al. et al., 2018). Appropriate exercise may reduce the damage to myocardial cells from a high glucose environment by improving the substrates of mitochondrial oxidation. Running training significantly improved lipid deposition and FAO damage induced by diabetes, and played a positive role in the recovery of myocardial mitochondrial function. FGF21 plays an essential role in regulating this process (Jin et al., 2022). Broderick et al. (2005) performed 10‐week incremental treadmill training with DCM rats. After exercise intervention, it was found that the disorder of myocardial glucose and lipid metabolism in rats was improved, and cardiac function was also effectively restored. Hafstad et al. believed that compared to moderate‐intensity exercise, only high‐intensity exercise can alter the rate of myocardial energy substrate utilisation in normal rats. To test the effect of different exercise intensities on diabetic heart failure, 8‐week high‐intensity intermittent exercise (85–90% ${\dot{V}}_{O_{2}\max}$) and moderate intensity treadmill exercise (65–70% ${\dot{V}}_{O_{2}\max}$) were used. Both practices were shown to increase myocardial glucose utilisation in rats, reduce FFA levels and improve the body's aerobic capacity, although there was no change in lipid metabolism (Hafstad et al., 2013). Wang et al. (2020) found that after 16 weeks of moderate‐intensity aerobic exercise training, the increased mitochondrial membrane potential in the diabetic myocardium, increased mitochondrial oxidative capacity in the myocardium, and activation of the AMPK/PGC‐1α pathway promoted the transformation of myocardial energy metabolism from fatty acid oxidation to glucose oxidation, the maintenance of cardiac metabolic homeostasis, as well as a reduction in oxidative stress. Physical activity effectively improves the utilisation of myocardial mitochondrial energy substrates, but the mechanism is unclear. The effects of different forms of exercise on disorders of myocardial mitochondrial lipid and glucose metabolism should be further verified.

Under normal physiological conditions, the myocardial tissue can scavenge ROS generated during metabolic activities through endogenous, enzymatic and non‐enzymatic antioxidant systems to protect the myocardial system from oxidative stress. However, in a high‐glucose environment for an extended period, as the antioxidant system of the myocardium is destroyed, changes in mitochondrial oxidative substrates lead to an increase in the production of oxidative substances such as ROS, which leads to the generation of mitochondrial oxidative stress in the myocardium. Several studies suggest moderate‐intensity exercise can reduce ROS generation, improve myocardial mitochondrial function, and reduce oxidative stress‐induced injury (Ma et al., 2021). Diabetes in mice leads to severe oxidative stress in the heart, which is linked to perturbations in ATP production and energy metabolism caused by an impaired mitochondrial respiratory chain in diabetic mice. Still, exercise intervention could reverse this and increase ATP synthesis. In addition, the authors believe that running exercise induces a significant up‐regulation of cardiac klotho β (KLB) protein (the co‐receptor of FGF21), which makes cardiomyocytes susceptible to the effect of FGF21, and reverses diabetes‐induced myocardial mitochondrial dysfunction, oxidative stress and fibrosis by stimulating mitochondrial SIRT3, thereby preventing or slowing down the process of DCM (Jin et al., 2022). Endurance aerobic exercise training can reduce the expression of the key regulatory subunits NADPH oxidase p47 (Phox) and p67 (Phox) in the hearts of diabetic rats and reduce the ROS generated during oxidative phosphorylation of mitochondria in the myocardium, effectively enhancing myocardial function (Sharma et al., 2016). Tumour necrosis factor α (TNF‐α) protein expression, as well as interleukin (IL)‐6 and IL‐1β mRNA expression, can be significantly reduced in the myocardium of *db*/*db* diabetic mice after 8 weeks of moderate‐intensity treadmill exercise, an increase in the level of eNOS, as well as an improvement in the symptoms of myocardial inflammation, insulin resistance and oxidative stress (Broderick et al., 2019). It is also possible that aerobic exercise inhibits mammalian sterile 20‐like kinase 1 (Mst1) activity via the Akt pathway, decreases oxidative stress in the myocardium of diabetic mice, decreases the formation of mitochondrial ROS, reduces mitochondrial swelling, promotes mitochondrial ATP production and increases mitochondrial membrane potential levels (Zhao et al., 2020). Activation of the renin–angiotensin–aldosterone system mediates the up‐regulation of NADPH oxidase activity in the setting of insulin resistance and hyperglycaemia, activates the transforming growth factor β 1 (TGF‐β1)/Smad2/3 signalling pathway, promotes cardiac fibrosis and then has an effect on myocardial oxidative stress (Murdoch et al., 2014). Long‐term moderate intensity exercise can inhibit the myocardial TGF‐β1/Smad2/3pathway, reduce oxidative stress and improve myocardial fibrosis in T2DM rats (Wang et al., 2019). On the other hand, a high glucose environment stimulates adipocytes to continuously produce inflammatory cytokines, for example, TNF‐α, IL‐6 and so forth (Lu et al., 2020). FFA and ROS induced by electron uncoupling can also directly stimulate proinflammatory cytokine production through the activation of nuclear factor‐κB (NF‐κB) and aggravate the inflammatory response, leading to the induction of an increase in oxidative stress (Al‐Rasheed et al., 2017; Li et al., 2019). Physical activity may protect mitochondrial function by reducing the production of oxidatively active substances and by increasing mitochondrial oxidative stress.

## 4 CONCLUSION AND PERSPECTIVE

Myocardial mitochondrial dysfunction induced by a high‐glucose environment is one of the leading causes of DCM. Primary mitochondrial mechanisms include mitochondrial metabolic substrate disorder, altered mitochondrial dynamics, mitochondrial Ca^2+^ regulation disequilibrium, microRNA regulation defect, mitochondrial oxidative stress, and so forth. Exercise can promote myocardial mitochondrial biogenesis, autophagy level, fusion and division balance, improve oxidative stress, and improve myocardial mitochondrial metabolism through PGC‐1α, SIRT3, FGF21 and other signalling pathways to resist DCM.

Further optimisation of the appropriate training format, training intensity, duration, frequency of training, and other training programs is needed to determine the effect of exercise on DCM. Future studies should pay more attention to the role of miRNAs and cellular movement factors in DCM intervention that is mediated by different types and modes of exercise. The imbalance of mitochondrial Ca^2+^ regulation is the primary mechanism of DCM, but there are few studies on improving myocardial mitochondrial Ca^2+^ regulation by exercise. In the future, we should pay more attention to research in this field.

## AUTHOR CONTRIBUTIONS

Zhang Feng, Wang Jun and Lin Jianjian designed the concept of this article and drafted the manuscript, and prepared the figures. Zhang Feng, Wang Jun and Tian Haonan contributed to the revised edition. All authors have read and approved the final version of this manuscript and agree to be accountable for all aspects of the work in ensuring that questions related to the accuracy or integrity of any part of the work are appropriately investigated and resolved. All persons designated as authors qualify for authorship, and all those who qualify for authorship are listed.

## CONFLICT OF INTEREST

No competing interests declared.
